# Advancing well-being in clinical and translational science

**DOI:** 10.1017/cts.2023.709

**Published:** 2024-03-20

**Authors:** Miriam A. Bredella, Lunthita M. Duthely, Darshan H. Mehta, Gretchen Roman, Susan Pusek, Tara G. Bautista, Munziba Khan, Jessica P. Meyer, Alfred Vitale

**Affiliations:** 1 Massachusetts General Hospital and Harvard Medical School, Boston, MA, USA; 2 NYU Langone and Grossman School of Medicine, New York, NY, USA; 3 University of Miami School of Medicine, Miami, FL, USA; 4 University of Rochester Medical Center, Rochester, NY, USA; 5 North Carolina Translational and Clinical Sciences Institute, Chapel Hill, NC, USA; 6 Northern Arizona University, Flagstaff, AZ, USA; 7 National Center for Advancing Translational Sciences, National Institutes of Health Bethesda, MD, USA; 8 Stanford University, Stanford Medicine, Spectrum, Stanford’s Center for Clinical and Translational Research and Education Stanford, CA, USA; 9 University of Rochester Clinical & Translational Science Institute, Rochester, NY, USA

**Keywords:** Well-being, education, clinical and translational science award (CTSA), research training, clinical and translational research

Before the COVID-19 pandemic, a growing body of literature documented how provider well-being and perceptions of burnout threatened healthcare delivery. Similar reports were also emerging about the same issues affecting entry and retention into the biomedical research workforce. The COVID-19 pandemic further accelerated these conversations and documented a worldwide healthcare and research workforce crisis, marked by elevated levels of anxiety, depression, and burnout, surpassing pre-COVID-19 levels. The unprecedented circumstances of the pandemic were a call to action to develop a comprehensive approach to well-being across training stages and job roles. This themed issue seeks to provide a venue for identifying and improving well-being concerns across different stages and roles within the translational science workforce and to disseminate effective interventions. Notably, the manuscripts document the pervasiveness of concerns about well-being and how diverse institutions and fields are investing in efforts to intervene at individual and institutional levels. The manuscripts in this issue share programs, concepts, results, and lessons learned that may help institutions implement well-being initiatives supporting scientists.

Pololi *et al*. [1] report on the perspectives of mid-career research and medical faculty. This manuscript examines not only levels of burnout but also vitality, flourishing, and other factors that predict the intention to leave academic medicine. The study is drawn from an ongoing parent study based in the USA of a diverse sample of mid-career physician investigators and Ph.D. scientists. The authors have taken an interesting perspective where they measured identity self-awareness and valuing differences and examined the data by representativeness. Forty physicians and Ph.D. participants enrolled in a faculty development program were compared to a matched sample (approximately 1:2.5). The majority of participants reported low mentoring quality and when coupled with perceptions of poor inclusion and trust predicted the intention to leave academic medicine. This work also reported on differences by underrepresentation, gender, and degree. Their findings recommend ongoing mentor training and highlight the need for continued training of mid-career physicians and scientists, to retain them and mitigate inequities they may propagate.

The Barr *et al*. [2] manuscript focuses on the nursing workforce. It introduces the concept of innovation competencies to enhance the ability of nurses to respond to the challenges of their jobs. Integrative Nurse Coaching is a 1-year faculty fellowship and coaching program to build innovation competencies, such as resiliency, creative thinking, and teamwork to enhance well-being. The program employed three different frameworks to shape the intervention: the Theory of Integrative Nurse Coaching guided all monthly small group and individual coaching; the Holistic Transcendental Leadership Model for Enhancing Innovation, Creativity, and Well-being in Healthcare was the basis for the program’s learning activities and training; and the Innovation Competence Model was used to design innovation competency conversations. The research team from the College of Nursing at The Ohio State University conducted individual interviews with the participants near the end of the program. Participants expressed enhanced comradery, increased confidence and ability to manage and prevent burnout, and shifted from a fixed to a growth mindset.

The manuscript by Patel *et al*. [3] addresses another workforce audience: physical therapists. The authors evaluate the impact of mindfulness-based training (MBT) on physical therapists’ well-being and burnout levels. In this mixed-methods pilot study, Patel et al. examined the effects of six MBT sessions over three months. Results indicated that physical therapists in the intervention group experienced improvements in work engagement, mental health, and moral distress. Challenges such as difficulty affecting change and prioritizing self-care were also identified. These findings provide the basis for future studies of an MBT program to enhance the well-being of physical therapists, potentially leading to improved employee retention and better patient care.

Turning to programs that aim to support the well-being of a broader audience, Shroff and Mehta [4] report on a novel solution to address the intensified stress levels during the pandemic across the workforce. They explore the Wellbeing Convene during COVID-19, a pilot program implemented at an academic Canadian institution. The program aimed to enhance the well-being of staff, faculty, residents, and students, fostering a sense of community during isolating times. Results indicate that participants found the program beneficial, leading to a recommendation for the permanent availability of such initiatives at no cost. However, the article underscores that while wellness programs are valuable, they alone cannot address the underlying causes of mental and emotional stress, which often stem from financial concerns, hierarchical workplace structures, and the nature of the work itself.

Another novel intervention is described by Li *et al*. [5] in a brief report on a program to support physician parents. Li et al. summarize findings on implementing a program (SOPPort) to improve wellness and productivity and reduce burnout amongst new parents and parents of young children. The program, which included lactation support, taking time from work and returning to work, was implemented over two years. Preliminary results were that the program was helpful and well-received by the participants.

Olsen *et al*. [6] describe an institutional program focusing on grants to provide well-being training and promote retention. The Massachusetts General Hospital’s Office for Well-Being created a Well-Being Education Grants program to encourage faculty and trainees to pursue training that fosters well-being. With a cap of $750 per grant, interested applicants were asked to explain their requested well-being activity and the anticipated benefits. Initially, the call for applications was three times per year, covering well-being activities over four months, but this later evolved to two times per year for six months. At the end of the activity cycle, recipients strongly agreed that the grant impacted their well-being and enhanced their work-life balance. When asked to report how the grant impacted them openly, recipients shared positive sentiments about stress reduction, well-being/self-care, and social connection. This case example illustrates the importance of institutions being vested and providing the necessary resources and funding to cultivate well-being.

Turning to an intervention to support physical well-being, James *et al*. [7] summarize their findings of a mind-body/meditative movement (“Tai Chi Easy”) intervention designed to address overweight and obesity amongst midlife/older women. Although preliminary, this study is one of the first to measure the long-term effects of meditative movement on weight management. Using a single-sample, pretest-posttest design, James et al. found an overall improvement in sleep quality and emotional eating. Sleep quality, in turn, was related to perceived stress (psychological well-being) and mood disturbance (emotional well-being). James et al. also reported a significant relationship between sleep quality and self-compassion and a relationship between body awareness (e.g., recognition of bodily needs) and emotional eating.

Finally, we included two manuscripts that guide the clinical and translational science community in approaching future research in well-being. The scoping review by Bautista *et al*. [8] aimed to explore conceptual and operational definitions of well-being within occupational health. Conducted through a broad search using terms related to well-being and scale/assessment, the study identified 388 peer-reviewed articles meeting inclusion criteria. The review revealed substantial variation in how well-being is defined across these articles, with many studies needing a clear definition or connection between conceptual and operational aspects. A total of 158 well-being assessments were represented, underscoring the literature’s absence of consistent definitions and standardized measurements.

To promote rigor, reproducibility, and robustness in integrative health research, Mezuk *et al*. [9] proposed the Michigan Integrative Well-Being and Inequality (MIWI) Training Program for early-career behavioral/social scientists, clinical/health services researchers, and minority health researchers interested in learning the necessary methodological skills and analytic tools to understanding health disparities. They highlighted the importance of interdisciplinary team science to address the intersection of environmental, psychosocial, and biological contexts within an equity framework. In addition to providing a rationale for their work and the structure and components of MIWI, these authors from the University of Michigan also shared results from their process evaluation of the program’s first cohort of scholars. Findings suggested that MIWI fostered new supportive research collaborations. After participation, scholars reported a broadened research network and demonstrated growth in being interdisciplinary researchers and designing integrated health research.

There is growing awareness of well-being in the translational science workforce and the creation of a nurturing and supportive scientific community where researchers can thrive. The manuscripts in this issue provide insights on practical strategies and lessons learned to guide the next steps. We hope this themed issue motivates readers to build on the findings in these manuscripts to further develop, refine, and disseminate training and support programs that create a more resilient and representative clinical and translational science workforce. By investing the well-being, institutions can cultivate a resilient and passionate workforce that drives forward transformative discoveries to improve human health.
